# Sterilized Sponge for Capping Pulps

**Published:** 1889-07

**Authors:** T. H. Parramore

**Affiliations:** Hampton, Virginia


### STERILIZED SPONGE FOR CAPPING PULPS.

BY T. H. PARRAMORE, D.D.S., HAMPTON, VIRGINIA.

The use of antiseptics and germicides in general surgery led me, a few years ago, to adopt them in dental practice, especially in the treatment of exposed pulps. My method of treatment and the theory upon which it is based I described in a paper read at the meeting of the Southern Dental Association, at Old Point Comfort, Virginia, in 1887. I have had no reason to change my practice since then.

The rationale of treatment is to induce the exposed and irritated pulp to deposit "secondary dentine" at the point of exposure, and not at hap-hazard in any part of the pulp cavity, a propensity all of us have had reason to deplore. In this treatment sterilized sponge serves a double purpose: it acts mechanically, by its presence protecting the pulp against injury by the material used as a filling; for this purpose it is peculiarly adapted, both on account of its aseptic character and of its soft, yielding, non-irritant nature. In the second place, its porous character invites the dentine-forming germinal matter or protoplasm to gather in its meshes and form a covering of secondary dentine where it will protect this delicate pulp, and not, as is so often the case, form pulp-stones, which then become a source of irritation. That sponge does become the seat of deposition of secondary dentine, covering and protecting the nerve from thermal and mechanical injury and preserving the tooth in all its organic vital perfection, I have had good opportunity to prove.

Since I began the use of sterilized sponge (about three years ago) I have treated over one hundred and fifty cases; of this number I have only known of six failures. Nearly all of these cases are in the mouths of patients whom I see constantly, and who would report if there was trouble. Many of the cases I have filled permanently, and in every instance I have found the dentine sensitive when excavating. In almost every case the pulp was at first slightly unhealthy, as indicated by its red, inflamed condition and its propensity to bleed upon the slightest provocation. I have not, however, on that account, hesitated to cap at once, depending upon the use of bichloride of mercury, and no other treatment, before applying the sponge-graft and filling.

In filling, too much stress cannot be laid upon the necessity of thoroughness in every minutia of the operation, leaving nothing to chance. After partially preparing the cavity I apply the rubber dam and complete the preparation, avoiding, if possible, wounding the pulp, but do not allow an unavoidable wounding of the pulp to interfere with the thorough preparation of the cavity. Having my cavity carefully prepared and my dam adjusted, I next sterilize everything that is directly or remotely connected with the cavity, from this point to the completion of the operation; upon this point it is impossible for me to be too explicit or insist too earnestly, for on this more than anything else depends the success of the operation. Now place a small piece of sterilized sponge upon the point of exposure and fill over this with the oxyphosphate. There is no necessity for using the oxyphosphate very soft; no trouble need be feared from pressure upon the pulp; the sponge protects it against that.

When the cavity is deep enough to admit of it, I prefer to fill partially with oxyphosphate and finish the operation with gutta-percha, for two reasons: gutta-percha is a better protection against thermal changes than oxyphosphate; and, secondly, when this temporary filling is to be removed for the purpose of filling permanently, I have simply to remove the gutta-percha and find my cavity ready for the permanent filling. After allowing the temporary filling to remain for six months or more I fill permanently, allowing enough oxyphosphate to remain to protect the pulp.

